# Development of the United States Environmental Protection Agency’s Facilities Status Dashboard for the COVID-19 Pandemic: Approach and Challenges

**DOI:** 10.3389/ijph.2022.1604761

**Published:** 2022-05-24

**Authors:** Lisa Baxter, Jeremy Baynes, Anne Weaver, Anne Neale, Timothy Wade, Megan Mehaffey, Danelle Lobdell, Kelly Widener, Wayne Cascio

**Affiliations:** Office of Research and Development, United States Environmental Protection Agency (EPA), Research Triangle Park, NC, United States

**Keywords:** public health, COVID-19, occupational health, data visualization, geographic information system

## Abstract

**Objectives:** Develop a tool for applying various COVID-19 re-opening guidelines to the more than 120 U.S. Environmental Protection Agency (EPA) facilities.

**Methods:** A geographic information system boundary was created for each EPA facility encompassing the county where the EPA facility is located and the counties where employees commuted from. This commuting area is used for display in the Dashboard and to summarize population and COVID-19 health data for analysis.

**Results:** Scientists in EPA’s Office of Research and Development developed the EPA Facility Status Dashboard, an easy-to-use web application that displays data and statistical analyses on COVID-19 cases, testing, hospitalizations, and vaccination rates.

**Conclusion:** The Dashboard was designed to provide readily accessible information for EPA management and staff to view and understand the COVID-19 risk surrounding each facility. It has been modified several times based on user feedback, availability of new data sources, and updated guidance. The views expressed in this article are those of the authors and do not necessarily represent the views or the policies of the U.S. Environmental Protection Agency.

## Introduction

The severe acute respiratory syndrome coronavirus 2 (SARS-CoV-2) responsible for the pandemic spread of the coronavirus disease (COVID-19) was first identified in the United States (US) on 21 January 2020 in Washington State [1, 2] prompting the Centers for Disease Control and Prevention (CDC) to release guidelines for social distancing and masking in doctors’ offices. On 13 March, the President declared a national emergency [3] as COVID-19 outbreaks brought widespread local and state stay-at-home orders and shutdowns across the country.

Beginning in late March 2020, all United States Environmental Protection Agency (EPA) facilities were placed in a Federal Facility Business Continuity Plan (FFBCP) or a Continuity of Operations Plan (COOP) effectively mandating telework, closing facilities, and limiting facility access to essential personnel for critical operations. In the spring of 2020, the White House’s “Guidelines for Opening Up America Again” [4] and CDC’s “CDC Activities and Initiatives Supporting the COVID-19 Response and the President’s Plan for Opening America Up Again” [5] were released outlining criteria to consider when evaluating the readiness of a location to reopen while mitigating the risk of exposure to COVID-19 to returning staff. As the pandemic reached its second year and vaccines became available, updated Presidential Directives and CDC guidance were released to address the country’s changing needs [6].

Scientists in EPA’s Office of Research and Development developed the EPA Facility Status Dashboard (henceforth, the Dashboard), an easy-to-use web application that applies these guidelines to the EPA facilities located across the United States. It was first released internally to the Agency in June 2020 and has been modified several times based on user feedback and updated federal guidance. The Dashboard was designed to provide readily accessible information for EPA management and staff to view and understand the COVID-19 risk surrounding each facility. The objective of this paper is to describe the methods and approach to development the Dashboard.

## Methods

In this section we describe the overall scientific approach taken to develop the Dashboard including the data sources and analytical methods used through its various iterations.

### Delineating Commuting Areas

**FIGURE 1:**
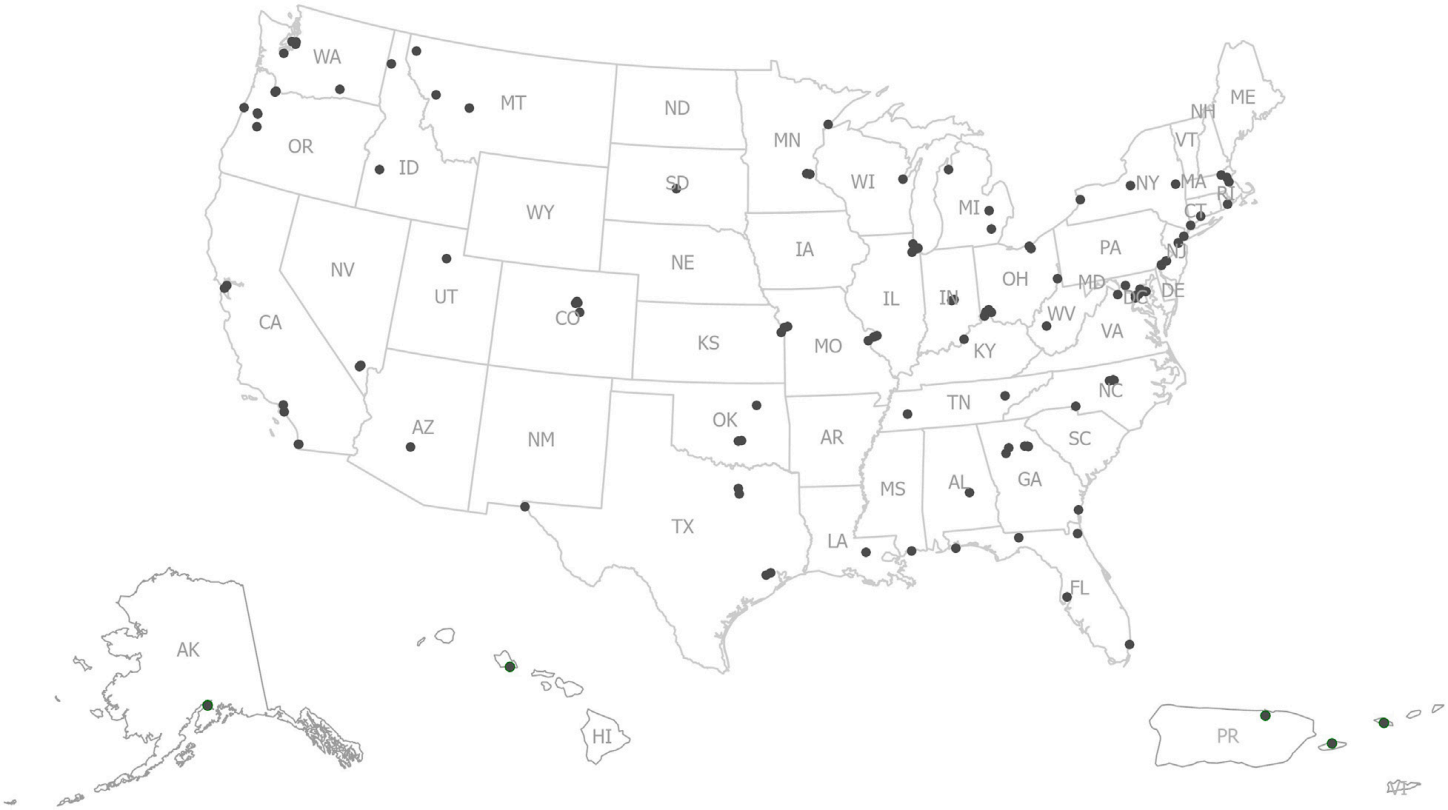
Map of United States Environmental Protection Agency’s Facilities (United States of America, 2020–2021).

The EPA has more than 120 locations across 40 U.S. states (Figure 1). Like many businesses across the country, EPA staff commute from counties and sometimes states other than the one where their facility is located. Recognizing the importance of the entire commuting area, rather than just the county where each facility is located, the team included the entire geographic extent of EPA employee residence areas for each facility. Since the team did not have access to employees’ home addresses, an estimate of likely commuting area for each facility was created using data from the U.S. Census Bureau’s 2015 American Community Survey (ACS) [7]. The ACS is a rolling survey that provides vital information on a yearly basis about our nation and its people. The Economic collection titled Commuting (Journey to Work) includes the 2011–2015 5-Year ACS Commuting Flows Tables that couple workers’ residences and workplace locations.

The county of each EPA facility was determined from latitude and longitude data and then paired with the ACS’s community flow table to select the most likely counties in the commuting area. Commuting counties were selected that provided at least 1% of the total commuters to the EPA facility’s county. Some independent cities (i.e., not part of a county) in the Washington, DC region were surrounded by counties identified in the commuting area, but did not meet the 1% threshold (e.g., Manassas, Falls Church). These cities were included to fill holes in the commuting area. The selected counties were grouped by facility to create a geographic information system (GIS) boundary for each EPA facility’s commuting area that is used for display in the Dashboard and to summarize population and COVID-19 health data for analysis.

### Data Sources

At the time of Dashboard’s initial development, there was no publicly- accessible, centralized data source that could provide all necessary data to assess county-level COVID-19 conditions. The team evaluated many sources of data and chose the most complete and geographically resolved ones for use in the Dashboard. The data sources changed and evolved over time as the response to the pandemic progressed. Table 1 presents all indicators and datasets, including those that were retired, that have been used in the Dashboard.

**TABLE 1:** Past and current indicators and data Sources for the Environmental Protection Agency Facilities Status Dashboard (United States of America, 2020–2021).

Indicators	Data source	Spatial and temporal resolution	Retired or active
Influenza-like Illness Symptoms	FluView (via ILINet^a^)	State-level and weekly	Retired as of 07/01/2020
	NSSP^b^ database (*via* HHS Protect)	County-level and daily	Retired as of 04/22/2021
COVID-like Illness Symptoms	NSSP^b^ database (*via* HHS Protect)	County-level and daily	Retired as of 04/22/2021
Cases	John Hopkins Coronavirus Resource Center	County-level and daily	Active as 05/27/2020
Percent Positive Tests	COVID Tracking Project	State-level and daily	Retired as of 07/01/2020
	HHS Protect	County-level and daily	Active as of 07/01/2020
ICU Capacity	NHSN^c^, AHA^d^, and TeleTracking (*via* GeoHealth Platform)	Hospital level and daily	Retired as of 07/01/2020
	NHSN^c^ and TeleTracking (*via* HHS Protect)	Hospital level and daily	Retired as of 04/22/2021
	http://www.healthdata.gov	County level and daily	Active as of 04/22/2021
COVID Hospitalization Admission Rates	http://www.healthdata.gov	County level and daily	Active as of 04/22/2021
Fully Vaccinated Rates	http://www.healthdata.gov	County level and daily	Active as of 04/22/2021

a ILINet: CDC’s Influenza-like Illness Surveillance Network.

b NSSP: CDC’s National Syndromic Surveillance Program.

c NHSN: National Healthcare Safety Network.

d AHA: American Hospital Association.

Initial data sources include Fluview, GeoHealth Platform, and the COVID Tracking Project for ILI/CLI ED visits, ICU capacity, and testing data, respectively. CDC’s Fluview [8] is an outpatient Influenza-like Illness Surveillance Network (ILINet) that provides ILI, but not CLI, data on a state and weekly basis. Data sources on the Department of Health and Human Services’s (HHS) GeoHealth Platform [9] include the National Healthcare Safety Network (NHSN) (a database operated by CDC to collect information on healthcare-associated infections), the American Hospital Association, and Teletracking (a private company that tracks hospital capacity). The COVID Tracking Project [10] is a volunteer organization dedicated to collecting and publishing daily state-level data required to understand COVID-19 outbreaks in the United States.

Beginning in July 2020, the team exported ILI, CLI, and percent positive test data via HHS Protect, a secure data platform and repository limited to authorized government employees and contractors created to support the White House Coronavirus Task Force [11]. Databases in HHS Protect include the National Syndromic Surveillance Program (NSSP) database, which includes both ILI and CLI data [12], NHSN (retired as a source to HHS Protect 14 July 2020); TeleTracking, and data collected by states. To note, while there are different types of diagnostic tests, the analysis for percent positive test was restricted to the polymerase chain reaction (PCR) tests for SARS-CoV-2 RNA, considered the gold standard for COVID-19 tests and used throughout the pandemic [13].

For tracking newly identified COVID-19 cases, the Johns Hopkins University (JHU) Coronavirus Resource Center data [14] was found to be the most readily accessible and regularly updated source of cumulative confirmed COVID-19 cases. JHU collects and analyzing daily domestic and international data about COVID-19 and tracks daily cumulative confirmed cases (including presumptive positive cases) by county for all 50 states, Washington DC, United States. Virgin Islands, and Puerto Rico.

Presently, Healthdata.gov [15] is the Dashboard’s source of data for ICU capacity, hospitalization admissions due to COVID, and COVID vaccination rates. Healthdata.gov is a federal government website managed by the U.S. Department of Health and Human Services.

### Gating Criteria Analysis

The White House’s “Guidelines for Opening Up America Again” [4] focused on three-gating criteria:

• Criterion 1: Symptoms—A downward trajectory of influenza-like illnesses (ILI) AND COVID-like illnesses (CLI), over the course of 14 consecutive days.
• Criterion 2: Cases—A downward trajectory of documented cases over a 14-day period OR a downward trajectory of positive tests as a percent of total tests over a 14-day period (flat or increasing volume of tests).
• Criterion 3: Hospitals—Ability and capacity to treat all patients without crisis care AND a robust testing program in place for at-risk healthcare workers, including emerging antibody testing.

To minimize the impact of daily fluctuations in the data, a three-day rolling average was used in all trend analysis. Trends were analyzed using an autoregressive model (SAS Version 9.4, Proc Autoreg) [16] with a *p*-value of less than 0.05 to indicate significance of upward or downward trends. With time series data, daily values are commonly correlated with each other making an autoregressive model the most appropriate type of model. Methods associated with each criterion follow.

For Criterion 1, the numbers of daily emergency department (ED) visits due to ILI and CLI symptoms and total ED visits were each summed for the counties included in each facility’s commuting area. The percentages of ED visits for ILI and CLI were calculated by dividing the daily number of ILI and CLI ED visits by the total daily ED visits and multiplying by 100. Once the data were compiled, an autoregression model was conducted for each facility for a 14-day time period ending on the Sunday before Dashboard updates. If the mean percent of ED visits for ILI over 14 consecutive days were below CDC’s region-specific thresholds [17], the facility was considered to have met the criterion, regardless of trend. The criterion was evaluated based on region-specific thresholds, but the national ILI minimal percentage (2.4%) was shown on the dashboard for illustrative purposes only.

There was no minimal threshold guidance from CDC for CLI levels, so the team adapted CDC methods [17] for calculating minimal ILI thresholds. CDC determined minimal ILI activity by calculating the mean percentage of patient visits for ILI during non-influenza weeks for the previous three seasons and adding two standard deviations,. As a surrogate for the “non-COVID” weeks, we assessed the mean percentage of patient visits for CLI for the 14 days ending on 1 July 2020 for counties with minimal (<10 per 100,000 over 14 days) confirmed COVID-19 cases and added two standard deviations. If the mean percent of ED visits for CLI over 14 days were minimal (currently set at 1.77%), the area was considered to have met the criterion, regardless of trend. This minimal percentage level was the same for all locations. This time period was chosen as it was the period between the spring and summer peak when incidence rates were at the lowest levels since the start of the pandemic.

Daily new cases, total numbers of tests and positive tests for the counties included in each facility’s commuting areas were summed to address Criterion 2. The percent of positive PCR tests for SARS-CoV-2 RNA was calculated by dividing the daily total of positive tests (summed over the commuting area) by the total number of tests performed and multiplying by 100. Autoregression models were conducted for each facility to determine trends for daily new cases and percent positive tests. A two-week incidence rate was calculated by summing the number of new cases for the commuting area and dividing that number by the total population of the commuting area and then multiplying that number by 100,000. A 14-day incidence of fewer than 10 cases per 100,000 population was considered a low-risk plateau in accordance with CDC Guidance [5]. Total number of tests and total number of positive tests were also reported in the Dashboard each week.

While the White House’s “Guidelines for Opening America Up Again” [4] state that the gating criteria are met if there is a downward trajectory of documented cases within a 14-day period OR a downward trajectory of positive tests as a percent of total tests within a 14-day period (flat or increasing volume of tests), CDC Guidance [5] provides further instruction on assessing this criterion. It states that “Laboratory test percent positive can be used in combination with, or as an alternative to, observing a decline in new case reports.” The team therefore chose to be conservative and designated the gating criterion as met if there was a downward trend of newly identified cases OR fewer than 10 newly identified cases per 100,000 over 14 days AND a downward trend in percent positive tests over 14 days (Table 2).

**TABLE 2:** Color threshold designations for the gating criterion (United States of America, 2020–2021).

Criterion	Color designation	Red (not meeting the criteria)
	Green (meeting the criteria)	
1	(CLI exhibiting downward trend for 14 days indicated by model trend and *p*-value < 0.05 OR minimal CLI activity) AND (ILI exhibiting downward trend OR minimal ILI activity)	Upward trend (*p* < 0.05) or no discernible trend (*p*-value ≥ 0.05) for past 14 days for either CLI or ILI.
2	(Downward trend of newly identified cases OR fewer than 10 newly identified cases per 100,000 over 14 days) AND (percent positive tests for 14 days indicated by model trend and *p*-value < 0.05, while not decreasing the overall number of tests	(Upward trend (*p* < 0.05) or no discernible trend (*p*-value ≥ 0.05) for newly identified cases OR 10 or more cases per 100,000 over 14 days) OR (upward trend in percent positive tests for past 14 days indicated by model trend and *p*-value < 0.05 or no discernible trend (*p*-value ≥ 0.05), while not decreasing overall number of tests)
3	Phase 1: At least 20% of ICU beds are available in the commuting area AND at most 20% of tests are positive for 14 days	Phase 1: Less than 20% of ICU beds are available in the commuting area OR more than 20% of tests are positive for 14 days
	Phase 2: At least 25% of ICU beds are available in the commuting area AND at most 15% of tests are positive for 14 days	Phase 2: Less than 25% of ICU beds are available in the commuting area OR more than 15% of tests are positive for 14 days
	Phase 3: At least 30% of ICU beds are available in the commuting area AND at most 10% of tests are positive for 14 days	Phase 3: Less than 30% of ICU beds are available in the commuting area OR more than 10% of tests are positive for 14 days
Overall	All criteria are met	Not all criteria are met

Criterion 3 required several types of information from localized sources that were not readily available. Based on CDC Guidance [5], the percent of intensive care unit (ICU) beds available was suggested as a measure for the first part of Criterion 3 (treating all patients without crisis care) and daily percent positive tests was recommended as an indicator for a robust testing program by indirectly measuring the agreement between testing demand and testing availability. ICU capacity was calculated at the hospital level as (available ICU beds/total ICU beds) multiplied by 100 and then averaged over all hospitals in the commuting areas where there was at least one ICU bed. Percent positive tests was calculated by summing across the counties included in the commuting areas as described for Criterion 2. The threshold designations for this criterion changed depending on what phase the facility was in (Table 2).

EPA developed a three-phased approach that provided for a “rolling reopening” for each facility that progressed through the phases based on the gating criteria and guidance from state and local public health authorities. The three phases for reopening included a range of telework and work schedule flexibilities, reduced staff and visitor access to buildings, and work travel restrictions. Guidance on day-to-day procedures also included alignment with local orders (ex. regarding face covering), social distancing requirements, and contact tracing.

### Levels of Community Transmission Analysis

Based on updated guidance [18–20], the current iteration of the Dashboard focuses on informing staff and management on the level of community transmission as defined by CDC based on certain thresholds as opposed to trends. Calculations of incidence rates, percent positives, daily new cases, and ICU capacity are the same except the time scale is now 7-day as opposed to 14 days ensuring consistency with the CDC guidance. The ILI and CLI symptoms indicators were removed during the most recent update of the Dashboard as they were no longer included in the update guidance. Additional indicators added include COVID hospital admission rates and fully vaccinated rates. Hospitalization rate is the percentage of inpatient beds occupied by COVID patients averaged over 7 days across the counties in the commuting area. The fully vaccinated rate is calculated by summing the number of residents in the commuting area that have been fully vaccinated divided by the population of the commuting area multiplied by 100.

## Results

**FIGURE 2:**
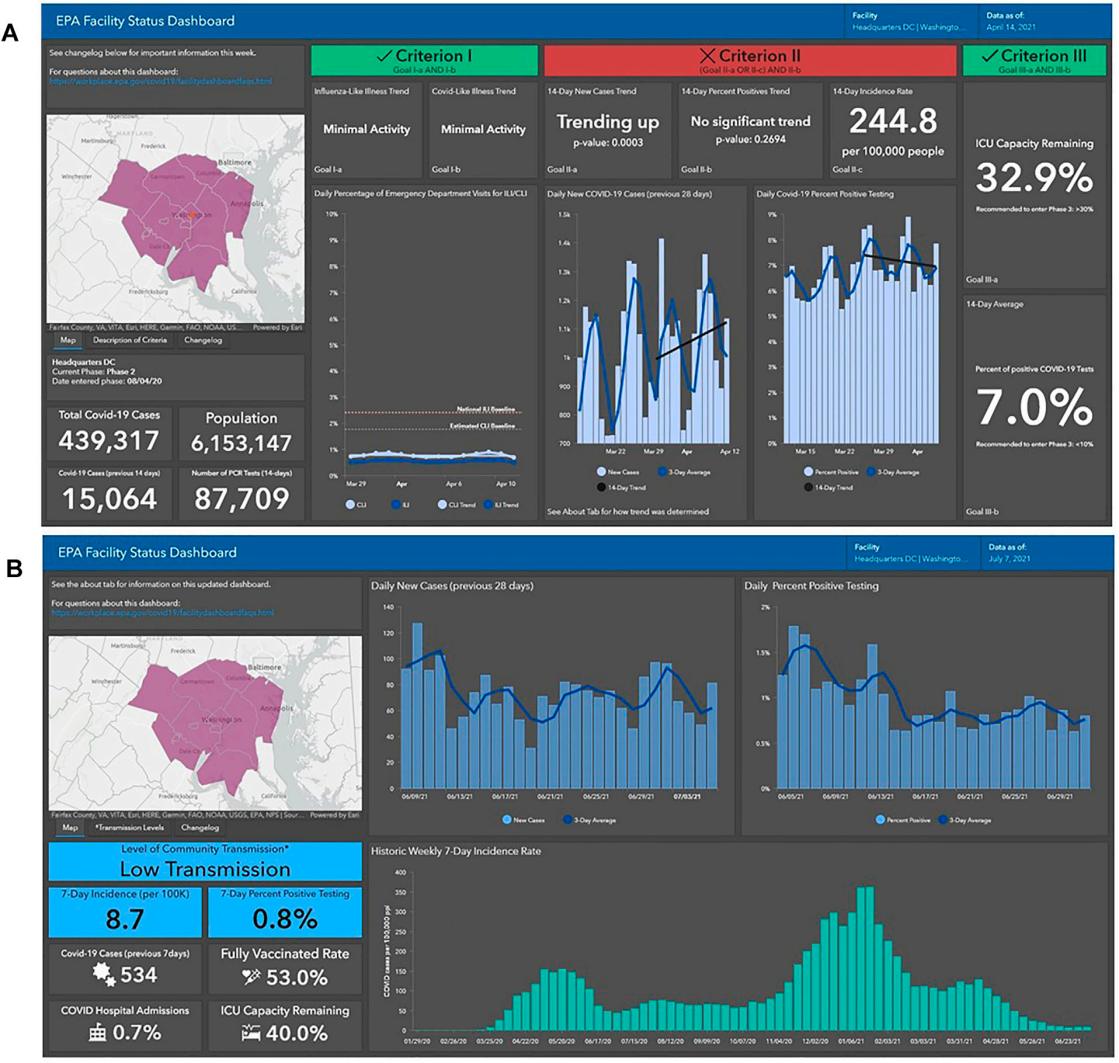
The Dashboard Facility View of the United States Environmental Protections Agency’s Headquarters using the three gating criteria **(A)** and levels of community transmission **(B)** (United States of America, 2020–2021).

The “EPA Facility Status Dashboard” was developed to provide broadly accessible visualization of the COVID-19 data most relevant to Agency workplaces. The dashboard included plots of the past 28 days as well as statistical trend results. When the dashboard focused on the 3 gating-criteria, a color-coding scheme was initially used to designate whether a facility was meeting the different gating criteria. Figure 2A presents a screenshot for EPA Headquarters for the week of 14 April 2021. Green indicated that a facility has met the criteria while red indicated it has not met the criteria. There was also an overall coloring scheme of green and red if a facility met all criteria or not met all criteria, respectively. The threshold and color-coding designations are specified in Table 2.

The Dashboard transitioned to designating the level of community transmission as defined by the CDC [6] and moved from a 14-day to a 7-day reporting time-period in order to be consistent with CDC guidance [21]. Figure 2B is an example screenshot of the updated Dashboard. Transmission level is determined by the combination of 7-day incidence rate and 7-day percent positive testing. Table 3 presents the color scheme and different thresholds for each category. The current iteration of the Dashboard continues to present 28-day plots of daily new cases and percent positives but has eliminated plots of ILI and CLI-symptoms. A plot of the historic weekly 7-day incidence rate showing long-term trends of COVID-19 for each area has also been added along with hospital admissions and vaccinations.

**TABLE 3:** Levels of community Transmission^a^ (United States of America, 2020–2021).

Indicator—if the two Indicators suggest Different transmission levels, the higher level is selected	Low transmission (blue)	Moderate transmission (yellow)	Substantial transmission (orange)	High transmission (red)
Total new cases per 100,000 persons in the past 7 days	0–9.99	10–49.99	50–99.99	≥100
Percentage of NAATs^a^ that are positive during the past 7 days	0–4.99%	5–7.99%	8–9.99%	≥10.0%

a Source: https://covid.cdc.gov/covid-data-tracker/#county-view.

**FIGURE 3:**
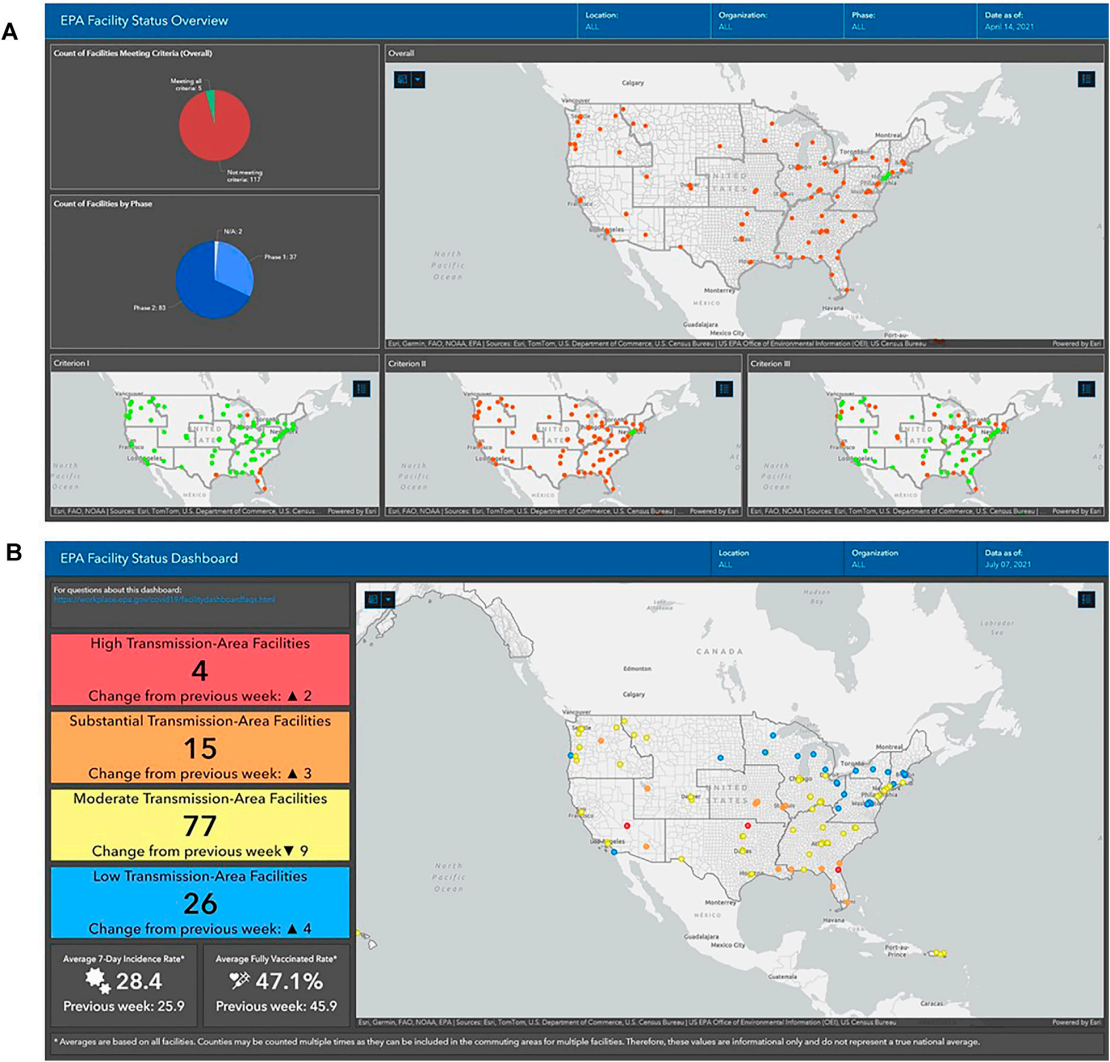
The Dashboard National View of all United States Environmental Protections Agency’s Facilities using the three gating criteria **(A)** and levels of community transmission **(B)** (United States of America, 2020–2021).

In addition to the “Facility-Specific View” where the user can select any EPA facility, there is also an option for “National View” that displays all EPA facilities in a map. Users can sort on a variety of factors such as by State, what part of the organization the facility is in, as well as which facilities have met all gating criteria (Figure 3A), or levels of community transmission (Figure 3B). This “National View” also presents summary statistics, for example fully vaccinated rates, across all EPA facilities.

As an example of the differences in the results during post-peak and peak incidences rates Supplemental File S1 present screenshots of both the Facility-Views and National Views during 2021 spring post-peak (Supplementary Figures S1, S3) and 2021 summer peak (Supplementary Figures S2, S4) community transmission levels.

Daily fluctuations in COVID-19 reporting led to a decision to update the Dashboard no more than once per week. Fluctuations were often caused by low or no reporting over the weekend days leading to spikes in number of cases reported on Mondays. The models were run for a 7-day or 14-day period ending on Tuesdays prior to Dashboard updates for new cases and symptoms. There was often a delay of several days between tests being ordered and results being reported so trends for percent positive tests were run using a 7-day and 14-day period ending on the Friday prior to the day the Dashboard was updated. All COVID-19 data were compiled, analyzed, and uploaded to the Dashboard on Wednesdays. Once the analyzed data were uploaded into the Dashboard, the information remained static until the following week. The user can view conditions for any week since the Dashboard’s release.

## Discussion

### Limitations of the Data

As described in the CDC publication [5], ILI is a non-specific syndromic overlapping with CLI symptoms indicator other respiratory illness. Additionally, the pandemic resulted in changes in health care seeking behavior, including the increased use of telemedicine, the recommendations to limit ED visits to severe illnesses, and the increased practice of social distancing. This likely affected the data reported from the networks supplying data, making it difficult to draw conclusions at the time.

There is considerable variability across states in the accuracy and timeliness of the reporting of the data. For example, not all locations report ICU capacity and that reporting varied by hospital and by state. We also encountered several challenges when working with case number and testing data. Some counties were not reporting or had limited reporting of new cases during weekend days, resulting in weekend lows followed by highs on Mondays or Tuesdays. Occasionally, large numbers reflecting positive or negative corrections to previous days were added or subtracted from an individual day resulting in outlier values. The outliers were reviewed weekly to determine whether there was a valid reason to remove the outlier or replace the value with data from another source (e.g., State COVID Dashboards). With rare exception, the value was not changed in the Dashboard. Outlier values were flagged if they were likely to have an impact on the trend. Another challenge with the cases data was that not all cases were reported strictly according to county boundaries and the analysis had to incorporate these exceptions. For example, although Kansas City is contained within multiple counties, the cases data were reported for Kansas City as a separate entity.

The HHS Protect testing data were limited in that there was a lag between the time of testing and the time results were reported. To adjust for the lag in test results, the team selected a 7-day or 14-day period ending on Friday of the previous week for the percent positive test trends. In addition, analyses used a 3-day rolling average rather than each day’s reported values in the autoregression analysis.

A significant amount of time was spent investigating and gaining access to various sources of COVID-related data. During the initial development of the Dashboard, there was no publicly available national database that contained all data necessary to evaluate individual facilities. For an organization distributed across the United States, like the EPA, it is time-consuming and labor intensive to go to the relevant websites of each of the 40 states where EPA facilities are to compile this data. Additionally, the data, may be stored, formatted, and curated differently by different states. HHS Protect does contain much of the data, but it is not publicly accessible, so the team had to apply for access. As we entered the second year of the pandemic, CDC’s COVID Data Tracker [6] created a national database (healthdata.gov) consisting of many of the required data elements, summarized by county, was made available to the public. This made data compilation more efficient.

### Data Interpretation

While guidance from the CDC assisted in assessing the gating criteria and levels of community transmission [5], there were still areas left open for interpretation. Many organizations could benefit from guidance that specifically pertains to re-opening places of business or other facilities. For example, for a business to re-open, it may be prudent for the decision makers to consider where their employees are coming from, rather than only considering the COVID statistics in the county where the business is located. The EPA Facility Dashboard team delineated a commuting area for each EPA facility to capture the COVID conditions of communities from which the majority of staff commute. Employees at EPA facilities come from a diverse background that include federal employees, interns, federal contractors, cafeteria, janitorial, facilities, and administrative staff that the team felt were well represented by the ACS Commuting Flow table. Other potential useful data such as SafeGraph (www.safegraph.com) was not freely available at the time of the Dashboard’s development.

Several aspects of the thresholds in the White House [4] and CDC guidance [5] lacked clarity. For example, Criteria 1 and 2 gating criteria are met if there are significant downward trends in symptoms, cases, and percent positive tests over a 14-day period. However, CDC guidance [5] specified a minimal activity threshold for ILI but not for CLI. The guidance states, “To pass the criteria of a 14-day downward trajectory in CLI syndromic cases, a locality must either have experienced 14 days of decreasing symptoms or exhibit near pre-pandemic levels of CLI”. However, no guidance was provided on what would define pre-pandemic levels. In addressing this guidance gap, the team developed a CLI minimal activity threshold for the Agency.

The CDC guidance [5] for Criterion 2 also included a low-incidence plateau of 10 new cases per 100,000 population over the 14-day period. There was no parallel lower threshold for percent positive tests—the guidance just states that this is met when there is a “near-zero percent positive.” The guidance also did not include upper thresholds for new cases or percent positive tests. A location may be experiencing downward trends in number of new cases and percent positive tests, but the incidence rate may still be extremely high, indicating widespread community transmission.

Revised CDC guidance [6, 21] addressed some of these limitations by focusing more on thresholds to define levels of community transmission as opposed to trends. While several scientific entities have recommended a variety of different phase thresholds, there is not a large body of scientific literature to support these thresholds. Testing and prevention strategies based on level of community transmission have been provided for K-12 schools, colleges and universities in a variety of CDC guidance documents [21]. However, for many other workplaces, each organization must develop their own approach to interpreting and implementing these strategies. It is good to allow organizations flexibility in how and if they allow workers back into their facilities, yet it may create confusion when approaches are not standardized.

### Communications of Results

Following the initial release of the Dashboard in March 2020, the team engaged in multiple webinars with EPA Regional and Program Office staff to highlight the Dashboard functions and capabilities, to detail the data sources used, and to explain how those data were being analyzed for each criterion. These webinars allowed the team to provide demonstrations, to respond to staff questions and concerns, and to receive feedback and input, some of which was later incorporated into the Dashboard, increasing its value. Additional communication actions included multiple agency-wide e-mails, agency-wide meetings among the different offices and teams, and the development of an intranet site updated with frequently asked questions and responses related to the Dashboard. The Dashboard itself includes contact information for immediate staff questions, an “About” tab that covers how the Dashboard was developed and how to interpret the visual graphics, and an area on the main page that documents any recent changes made to the Dashboard (for example, the incorporation of a new data source). These actions and feedback channels were implemented to ensure that the Dashboard remains dynamic and can adjust not only to evolving federal guidance, but also to the informational needs of the Agency staff.

### Conclusion

The EPA Facility Status Dashboard was designed to convey to the EPA leadership and staff the status of public health conditions within the commuting area of EPA facilities and to provide transparency around any reopening decisions. The graphical depiction of the burden of COVID-19 infection within these areas was modeled and adapted based on evolving federal guidance. Despite the challenges with data availability, interpretation, and communication, the EPA Facility Status Dashboard continues to serve an important role in informing management decisions and improving staff knowledge of the impact of COVID-19 in the communities surrounding EPA facilities. While the Dashboard was designed specifically to assess COVID-19 conditions surrounding EPA facilities, a visualization tool like the Dashboard could be useful to large multi-state organizations by presenting site-specific information in a way that is useful for decisions on returning to the workplace.
