# Supplementary material for: Development of the United States Environmental Protection Agency’s Facilities Status Dashboard for the COVID-19 Pandemic: Approach and Challenges
Source: Int J Public Health. 2022 May 24;67:1604761. doi: 10.3389/ijph.2022.1604761 (PMC9172581; doi:10.3389/ijph.2022.1604761)

Supplemental Figure 1. The Dashboard Facility View of the United States Environmental Protections Agency's Headquarters using levels of community transmission during spring post-peak (United States of America, 2021)

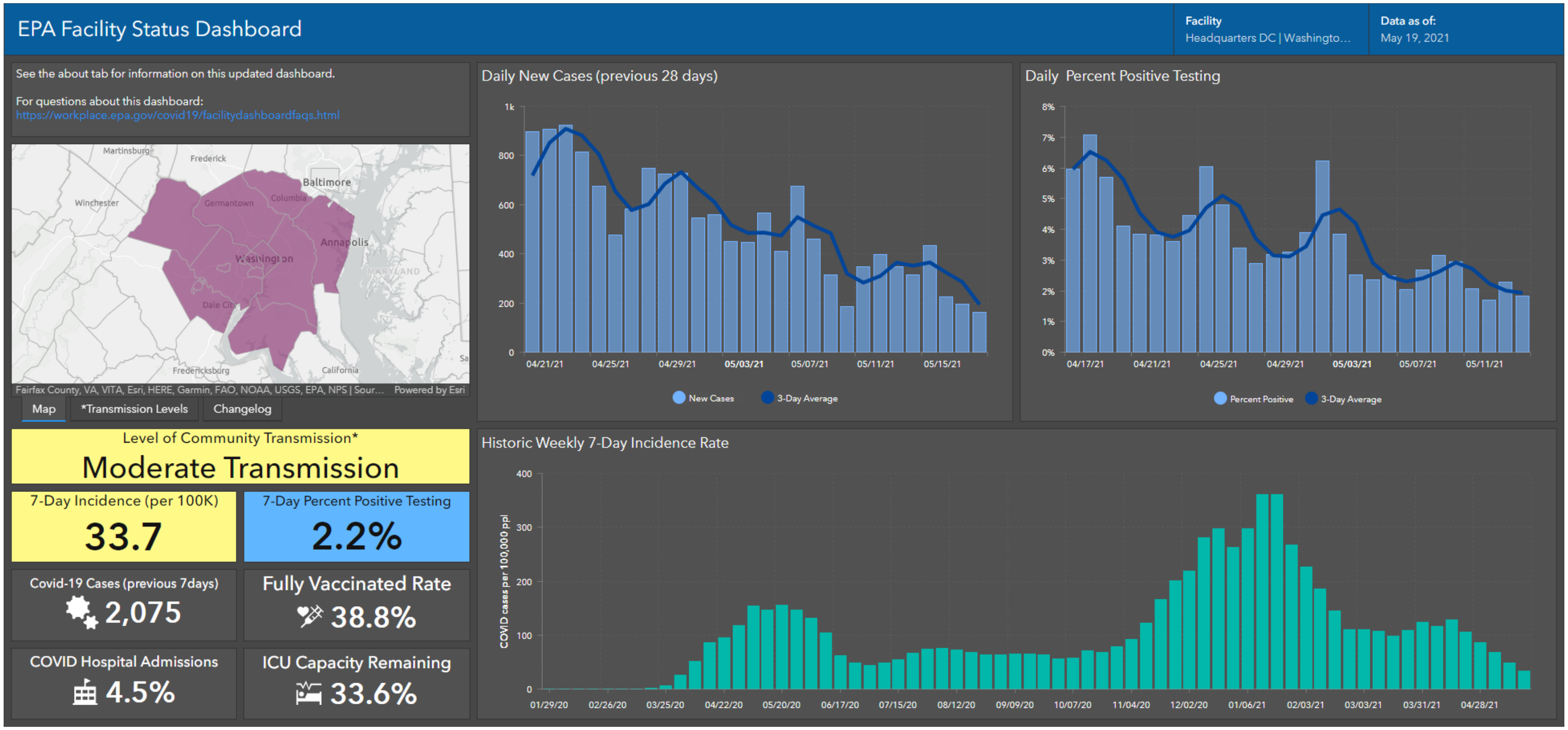

Supplemental Figure 2. The Dashboard Facility View of the United States Environmental Protections Agency's Headquarters using levels of community transmission during summer peak (United States of America, 2021)

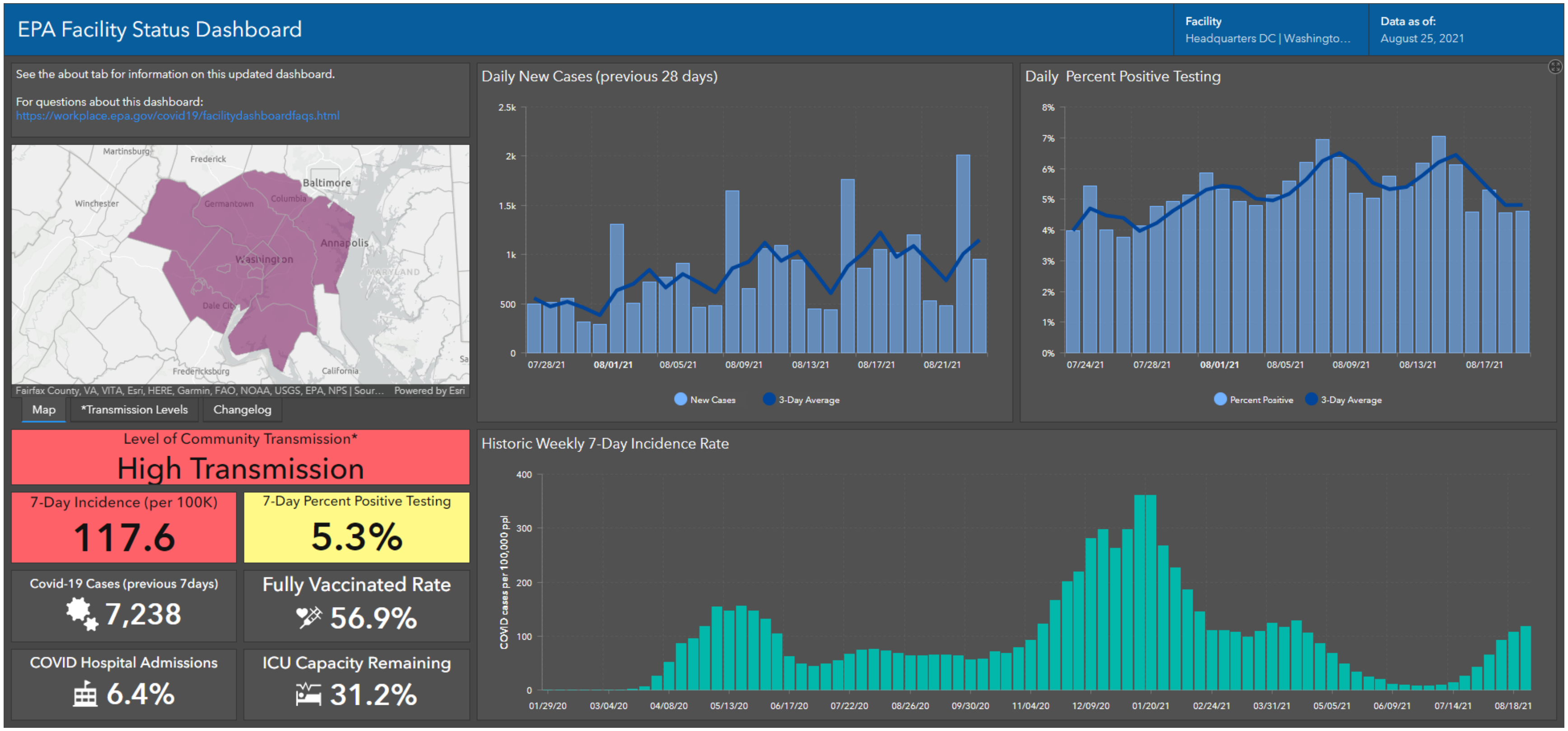

Supplemental Figure 3. The Dashboard National View of all United States Environmental Protections Agency's facilities using levels of community transmission during spring post-peak (United States of America, 2021)

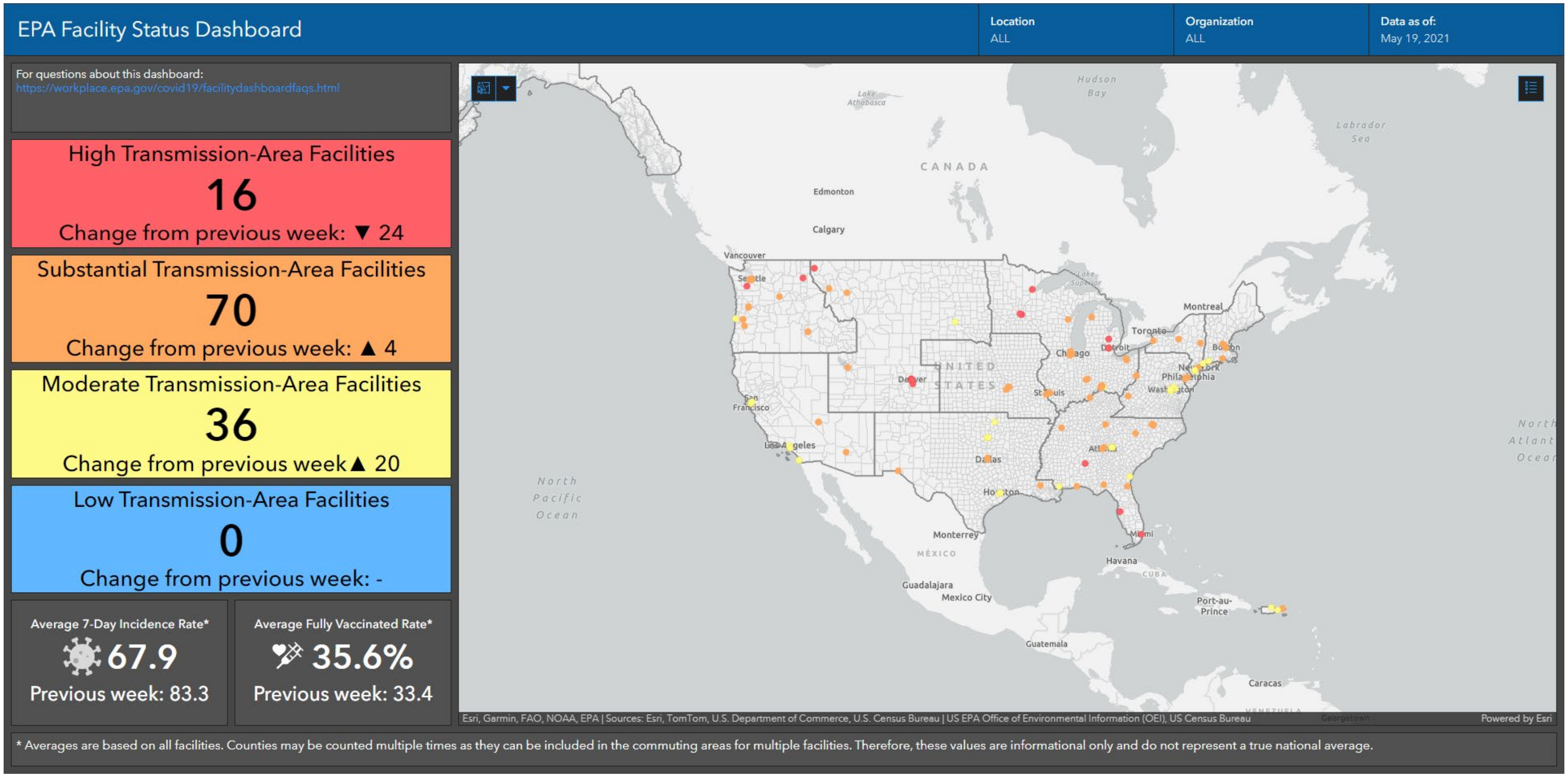

Supplemental Figure 4. The Dashboard Facility View of all United States Environmental Protections Agency's facilities using levels of community transmission during summer peak (United States of America, 2021)

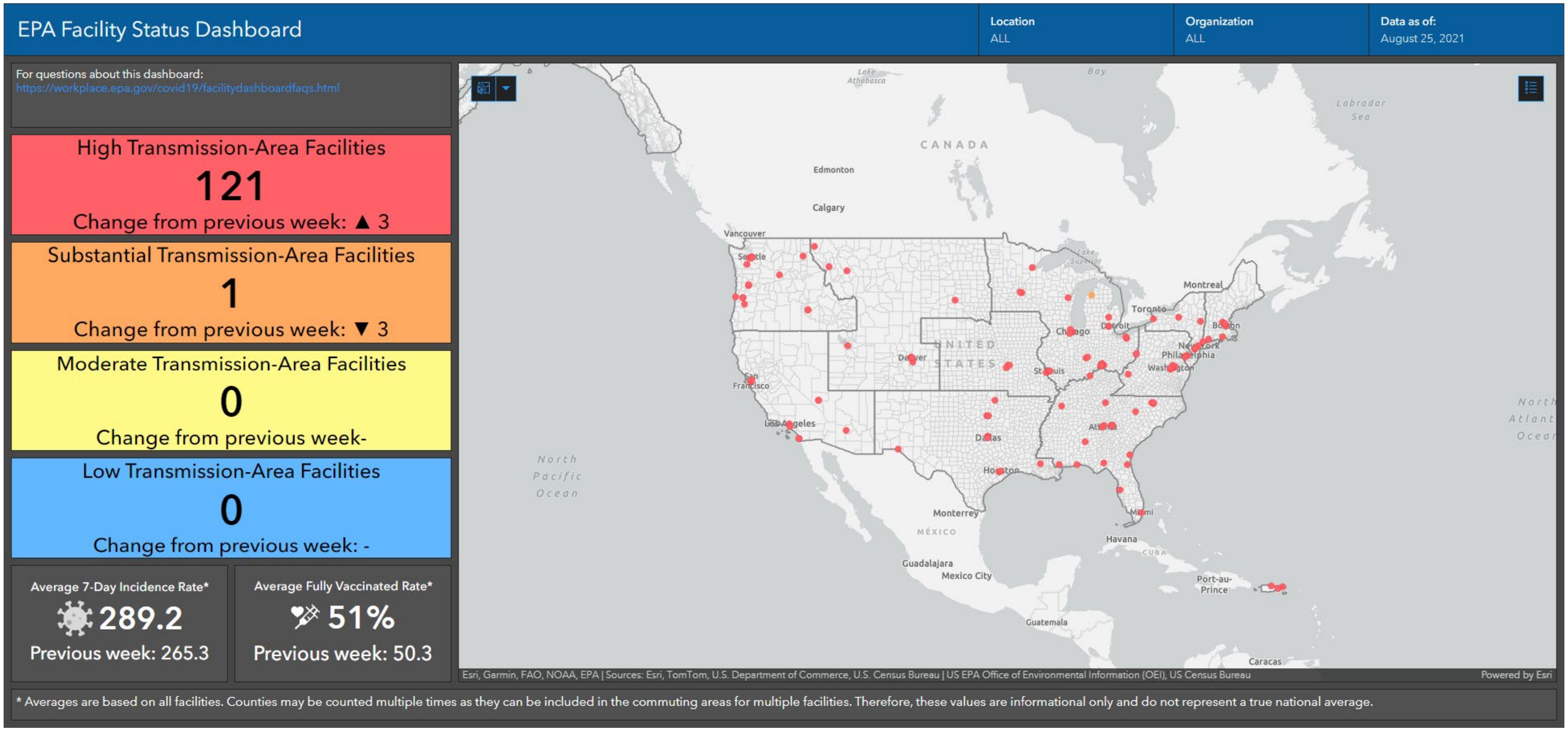

Supplement: Supplementary file 1 [file DataSheet1.PDF]
